# Delayed Wound Healing in Heat Stable Antigen (*HSA/CD24*)-Deficient Mice

**DOI:** 10.1371/journal.pone.0139787

**Published:** 2015-10-06

**Authors:** Shiran Shapira, Oded Ben-Amotz, Osnat Sher, Dina Kazanov, Jacob Mashiah, Sarah Kraus, Eyal Gur, Nadir Arber

**Affiliations:** 1 The Integrated Cancer Prevention Center, Tel Aviv Sourasky Medical Center, Tel Aviv, Israel; 2 Unit of Bone and Soft Tissue Oncology, The Institute of Pathology, Tel Aviv Sourasky Medical Center, Tel Aviv, Israel; 3 Department of Plastic and Reconstructive Surgery, Tel Aviv Sourasky Medical Center, Tel Aviv, Israel; 4 Department of Dermatology, Tel Aviv Sourasky Medical Center, Tel Aviv, Israel; 5 Sackler Faculty of Medicine, Tel Aviv University, Tel Aviv, Israel; Baylor University Medical Center, UNITED STATES

## Abstract

**Background:**

Healthy individuals rarely have problems with wound healing. Most skin lesions heal rapidly and efficiently within one to two weeks. However, many medical and surgical complications can be attributed to deficiencies in wound repair. Open wounds have lost the barrier that protects tissues from bacterial invasion and allows the escape of vital fluids. Without expeditious healing, infections become more frequent. The *CD24* gene encodes a heavily-glycosylated cell surface protein anchored to the membrane by phosphatidylinositol. *CD24* plays an important role in the adaptive immune response and controls an important genetic checkpoint for homeostasis and autoimmune diseases in both mice and humans. We have previously shown that overexpression of *CD24* results in increased proliferation and migration rates.

**Aim:**

To examine the role of *CD24* in the wound healing process.

**Methods:**

An excisional model of wound healing was used and delayed wound healing was studied in genetically modified heat stable antigen (*HSA*/*CD24*)-deficient mice (*HSA* ^*-/-*^) compared to wild-type (WT) mice.

**Results:**

Large full-thickness skin wounds, excised on the back of mice, exhibited a significant delay in the formation of granulation tissue, and in wound closure when compared to their WT*HSA* ^*+/+*^ littermates. Wounds were histologically analyzed and scored, based on the degree of cellular invasion, granulation tissue formation, vascularity, and re-epithelialization. Additionally, in stitched wounds, the *HSA* ^*-/-*^ mice failed to maintain their stitches; they did not hold and fell already 24 hours, revealing erythematous wound fields. Re-expression of *HSA*, delivered by lentivirus, restored the normal healing phenotype, within 24 hours post-injury, and even improved the healing in WT, and in BalbC mice.

**Conclusions:**

Delayed wound-healing in the absence of *HSA*/*CD24* suggests that *CD24* plays an important role in this process. Increased expression of *CD24*, even in the normal state, may be used to enhance wound repair.

## Introduction

Most skin lesions heal rapidly and efficiently within one to two weeks [1]. Although being healed, the outcome is neither esthetically nor functionally perfect [2]. In the U.S. alone, 35 million cutaneous wounds require major intervention annually [2].

Normal wound healing is a complex, dynamic and fragile process that is impacted by many factors and is divided into three phases, inflammatory, proliferative and maturation or remodeling [3,4]. After an initial wound, a fibrin clot is formed. In the inflammatory phase, debris and bacteria undergo phagocytosis and removal. Cytokines are released to initiate the proliferative phase. This process manifests with chemotaxis, phagocytosis, angiogenesis, epithelization, collagen degradation and remodeling, production of new glycosaminoglycans and wound contraction. Wound healing is a highly regulated interplay between systematic expressed cell types (i.e., neutrophils, macrophages, fibroblasts, keratinocytes, endothelial cells), extracellular matrix insoluble components and a group of soluble mediators (i.e., growth factors, cytokines, chemokines) [2,3,5].

The healing process begins with an accumulation of neutrophils and monocytes in the damaged tissue to form a first line of defense. Thereafter, macrophages and mast cells emigrate from nearby tissues and the circulation and accumulate in order to initiate the specific immune response. These inflammatory cells are recruited to the wound site by specific chemotactic factors or chemokines [6,7]. Re-epithelialization and granulation tissue formation include migration of cells from the wound edge to fill the wound site. It involves the migration of keratinocytes over the impermanent matrix in order to rebuild a protective layer [8].

Rapid changes in the extracellular matrix (ECM) occur during the healing process. The fibrin clot is replaced by fibronectin and hyaluronan and subsequently by type I and III collagen [1]. The contribution of each component to the wound repair process is difficult to assess due to the complexity of cells involved in the healing.

The *CD24* gene encodes a heavily glycosylated cell surface protein anchored to the membrane by phosphatidylinositol^6^. Human CD24 consists of 31 amino acids with 16 potential O-glycosylation and N-glycosylation sites. Owing to this extensive glycosylation, CD24 has mucin-like characteristics [9]. It plays a crucial role in cell selection and maturation during hematopoiesis and is expressed mainly on premature lymphocytes and certain epithelial and neural cells [10,11]. CD24 can function as an alternative ligand for P-selectin, an adhesion receptor on activated endothelial cells and platelets [12–14].

CD24 has been previously shown to play an important role in the inflammation [15] process. We also showed that overexpression of CD24 increased proliferation, and migration rates *in vitro* [16–18].

Herein, it is shown that CD24 plays a major role in wound healing, in HSA^-/-^ knockout (KO) mice as well as normal mice. We explored our theory that CD24(HSA) may be involved and contribute to the healing process. We examined whether there is a relationship between the mice genotype, HSA expression, and the healing process and rate. We confirmed our observations by immunohistochemistry (IHC) and collagen staining, and most importantly by re-expression of the *HSA* gene. We suggest here, for the first time, a new candidate for improving and accelerating the healing process.

## Materials and Methods

### Animal housing and procedures

Heat stable antigens (HSA) knockout (KO) mice on a C57BL/6J background were kindly provided by Prof. Peter Altevogt (DKFZ Heidelberg, Germany). Wild-type (WT)HSA^+/+^ mice were purchased from Harlan Laboratories (Rehovot, Israel). All mice were fed with a standard pellet diet and had access to tap water *ad libitum*. The animals were maintained at a constant temperature (22±2°C) on a 12 h light/dark cycle and all procedures were approved by the Institutional Committee for Animal Welfare at Tel-Aviv Sourasky Medical Center. Before the procedures, mice were anesthetized by intraperitoneal (i.p.) injection of ketamine (50 mg/kg) and xylazine (5 mg/kg). The dorsal surface of the animal was cleaned, shaved, and sterilized with a betadine solution. Longitudinal incisions, 1.5–4 cm, were made on the back of the mice. The excised wounds were left open, stitched, or dressed with gauze after virus administration. In HSA/mCherry expression experiments, the virus-containing medium or antibodies were applied once post-wounding. Wound healing was examined by macroscopic observations and histological analysis every day after wound excision. At the end of the experiments, the animals were sacrificed with a lethal dose of CO_2_.

### Virus production

Three HIV-based viral vectors (kindly provided by Prof. Eran Bacharach, Tel Aviv University, Israel); pVSV-G (5 μg), pCMV.ΔR8.2 (15 μg) and pHR’CMV-HSA (or pHR’CMV-mCherry, as negative control) (20 μg) were co-transfected to HEK293T helper cells using the standard calcium phosphate transfection method. hours after transfection, virions-containing supernatants were collected, the pH was adjusted with Hepes, filtered and stored at -80°C.

### Infection

Before the infection, polybrene was added to the virions-containing supernatant to a final concentration of 8μg/ml. The supernatant was added to NIH-3T3 cells, that were seeded in 6-cm plates the day before (5x10^5^ cells), for 2 hours. Then, 3 ml of fresh medium was added to the plate and after two days the infected cells were analyzed for the expression of the relevant gene.

In vivo, under anesthesia [intraperitoneal injection of ketamine (50 mg/kg) and xylazine (5 mg/kg)], 300 μl of viruses-containing medium were injected into the cells on the wound border or dropped directly into the wounds area of the mice. Wound closure and mice's well-being were monitored on a daily basis.

### *In vivo* targeting of *HSA* by anti-*HSA* mAb

Before the procedures, mice were anesthetized [ketamine (50 mg/kg) and xylazine (5 mg/kg)]. Longitudinal incisions, of 2 cm, were made on the back of the mice. Ten mg/kg of Rat anti-mouse M1/69 IgG2b mAb was administrated topically by injection into the cells on the wound border. Wound closure and mice's well-being were monitored every day.

### Imaging of mCherry expression in mice

For all imaging, mice were anesthetized using aketamine-xylazine mixture. For imaging of mCherry expression, mCherry-encoded viruses (300 μl of viruses-containing medium) were injected into the cells on the wound border or dropped directly into the wounds area of WT mice immediately after injury. The mCherry expression was monitored 96 hours later using the Maestro *in vivo* fluorescence imaging of whole small animals' device.

### Histology

At days 0, 3 and 14 days post-wounding, the animals were sacrificed with a lethal dose of CO_2_, autopsied, and their wound beds surrounded by a margin of non-wounded skin were collected. Samples were fixed with 4% paraformaldehyde overnight at room temperature, embedded in paraffin blocks, and sectioned. After deparaffinization and rehydration, the sections were washed and stained with Hematoxylin and Eosin (H&E) or NovaUltra^TM^ Picro-Sirius Red stain for collagen staining. Tissue sections were then washed, mounted, and visualized on an Olympus AH light microscope at 400× magnification.

### Protein extraction and immunoblotting

For protein extraction, cells were washed with PBS, scraped and lysed in 1%Triton buffer (100 mM NaCl, 5 mM EDTA, 1% triton, 50 mM Tris-HCl pH 7.5, 50 mM NaF, and a protease inhibitor cocktail (Roche), added just before use. Following 30 min of incubation on ice, lysates were cleared by centrifugation at 14,000 rpm for 10 min, at 4°C. For immunoblotting, protein samples were electrophoresed alongside a molecular weight marker on 10% SDS-Polyacrylamide gel electrophoresis (PAGE) and Western blot analysis was performed as described below.

### Western Blot analysis

Proteins resolved by SDS-PAGE were electro-transferred onto the nitrocellulose membrane. The membrane was blocked for at least 1 h with PBST containing 5% skim milk at room temperature (RT) with slow agitation. Proteins were detected using a specific primary antibody (M1.69, generous gift from Prof. Peter Altevogt; DKFZ Heidelberg, Germany) followed by horseradish peroxidase (HRP)-conjugated secondary antibody and enhanced chemiluminescence (ECL) detection using the EZ-ECL reagent as described by the vendor (Biological Industries, Beith HaEmek).

### Flow cytometry analysis

Cellular CD24 binding by rat M1.69 was evaluated by flow cytometry (FACS). Approximately 1x10^6^ cells were used in each experiment. After trypsinization, the cells were washed in fluorescence-activated cell sorting (FACS) buffer (10% FBS, 0.01% sodium azide in ice-cold PBS) and fixed with 2% formaldehyde for 15 min at RT. After washing with FACS buffer, 100 μl of 15 μg/ml anti-CD24 were added for 30 min at RT. After washing X3 with FACS buffer, fluorescein isothiocyanate (FITC)–labeled goat anti-rat (1:100) was added for 30 min at RT. Detection of bound antibodies was performed on a FACSCalibur (Becton Dickinson, San Jose, CA) and results were analyzed with the CELLQuest program (Becton Dickinson).

### Statistics

Data from the *in vivo* studies are presented as mean±SD of sets of data as determined for each group of mice. Statistical significant differences between groups and at different time points were determined by Student t-test, P values <0.05 were considered significant.

Wound areas were calculated by using the ImageJ software. Area selection was created and analyses for the areas were automatically done.

## Results

### Delayed wound healing in *HSA* KO mice

**Fig 1:**
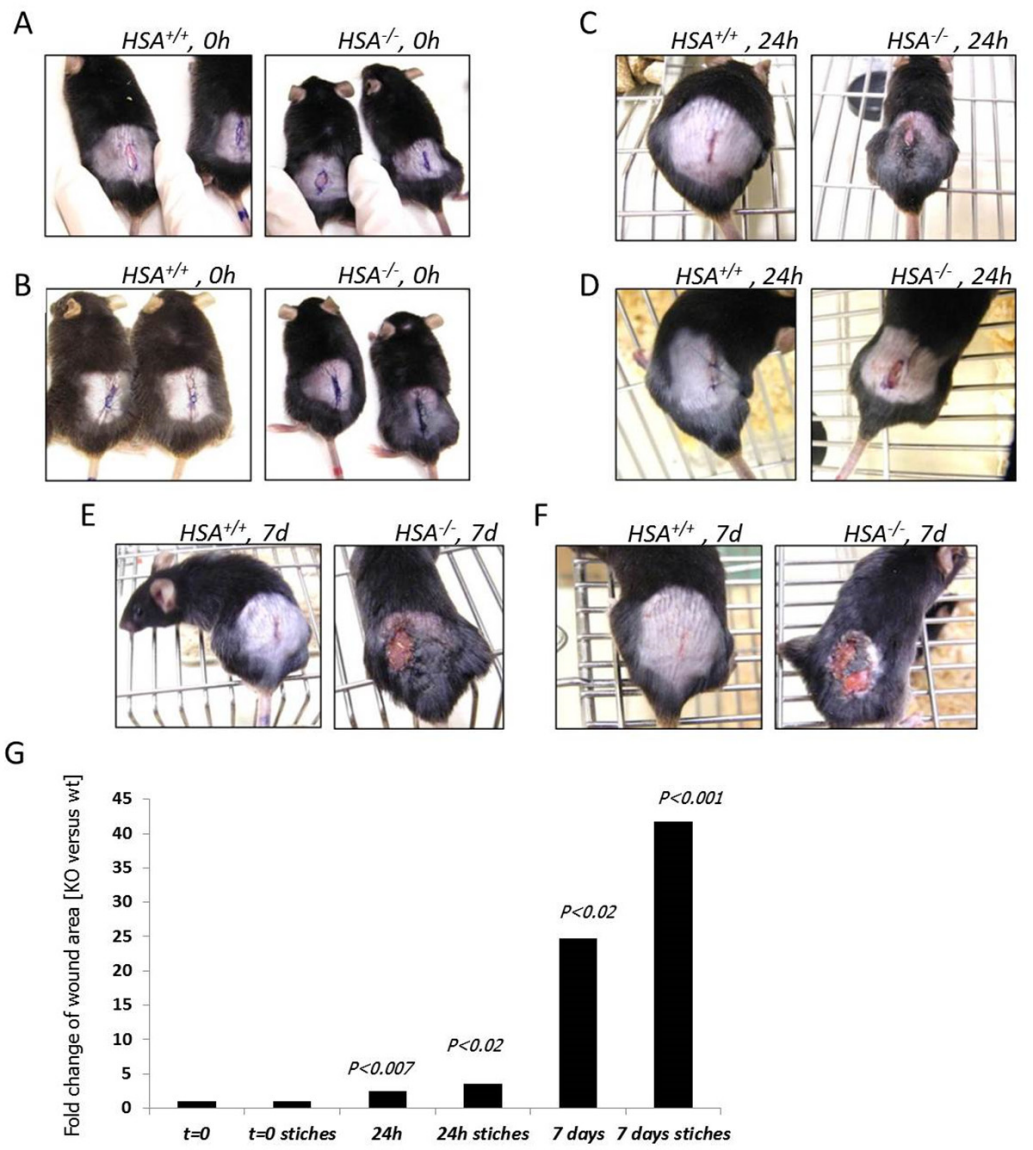
Wound closure of full-thickness wounds is delayed in *HSA* ^-/-^ mice. The left picture in each panel represents the *HSA* ^*+/+*^ mouse while the right is for the *HSA* ^*-/-*^. A. t = 0, time of injury. B. t = 0, the wounds were stitched upon injury. C. t = 24 h. D. t = 24 h, with stitches. E. t = 7 days. F. t = 7 days, with stitches. G. A representative plot of the statistical differences between the groups. Each bar shows the fold change of the wound area compared to t = 0.

**Fig 2:**
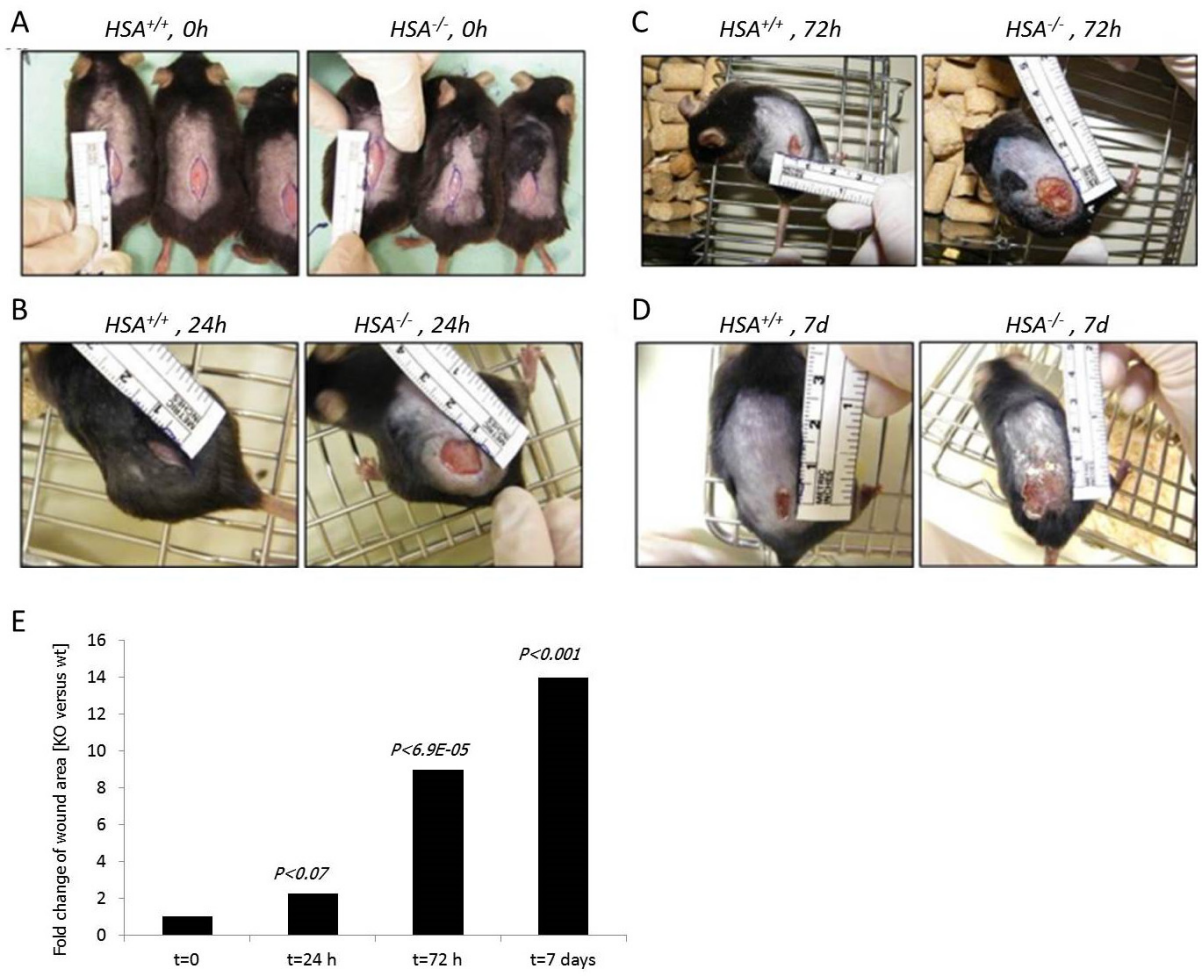
Wound closure of bigger full-thickness wounds is faster in *HSA* ^+/+^ mice. The left picture in each panel represents the *HSA* ^*+/+*^ mouse while the right is for the *HSA* ^*-/-*^. A. t = 0, time of injury. B. t = 24 h. C. t = 72 h. D. t = 7 days. E. A representative plot of the statistical differences between the groups. Each bar shows the fold change of the wound area compared to t = 0.

Several independent experiments with 10-mm oblong full-thickness excision wounds, including the striated muscle layer, on the dorsal skin of WT (*HSA* ^*+/+*^) (n = 5) and KO (*HSA* ^*-/-*^) (n = 5) mice demonstrated that the absence of HSA hampered the healing of skin wounds (Fig 1). Moreover, the KO mice failed to keep the stitches in the stitched wounds. Healing of KO mice wounds remained incomplete. In the HSA KO mice, scabs were thicker and erythematous wound fields were shown. Similar results were seen with longer excision wounds (n = 6) (Fig 2).

**Fig 3:**
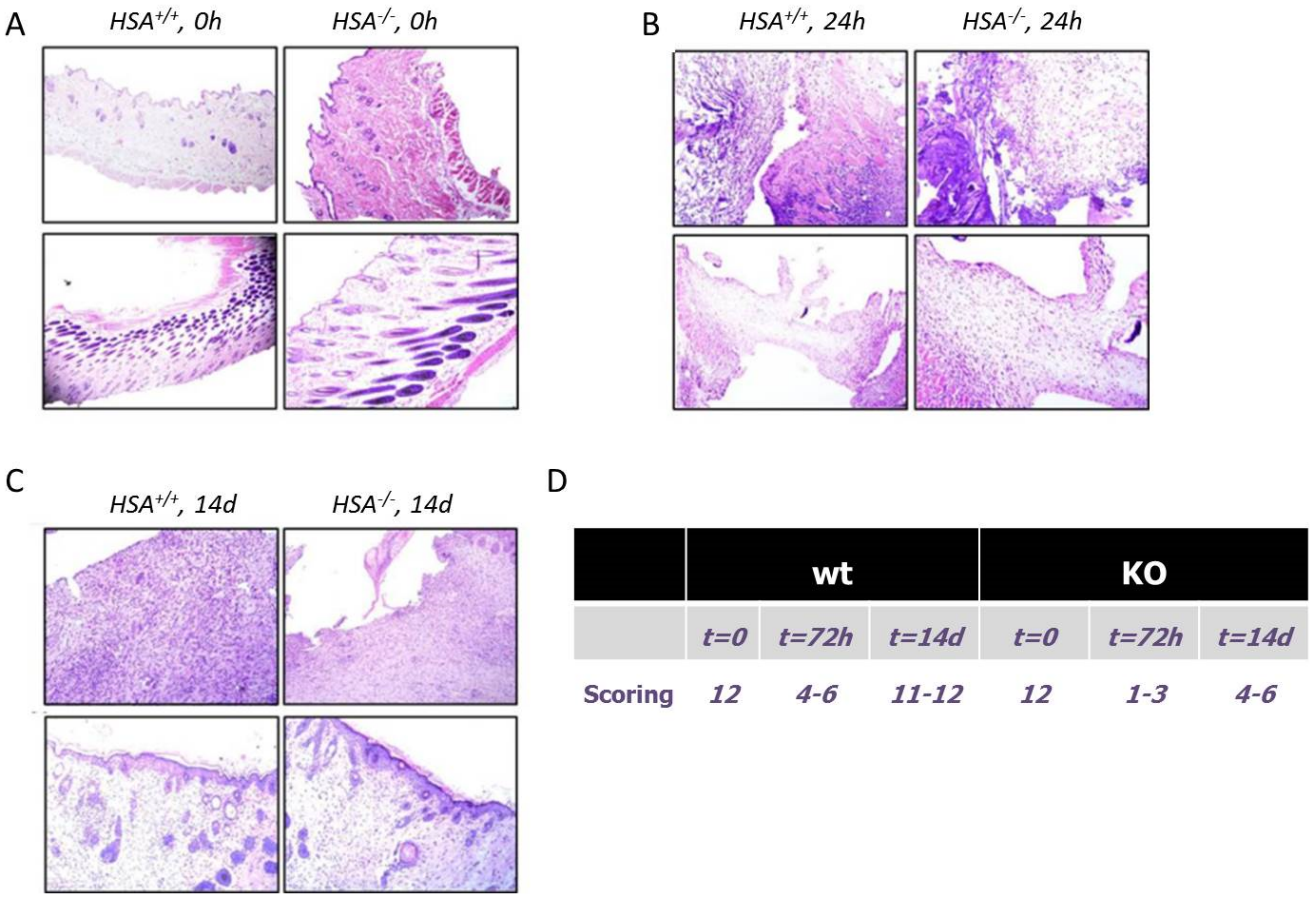
Histological stains. Tissue sections were stained with Hematoxylin and Eosin (H&E) and visualized on an Olympus AH light microscope at 400× magnification. The left picture in each panel represents the *HSA* ^*+/+*^ mice while the right is for the *HSA* ^*-/-*^. A- T = 0, time of injury; B- T = 72h; C- T = 2 weeks. D. Table of the scoring results.

**Fig 4:**
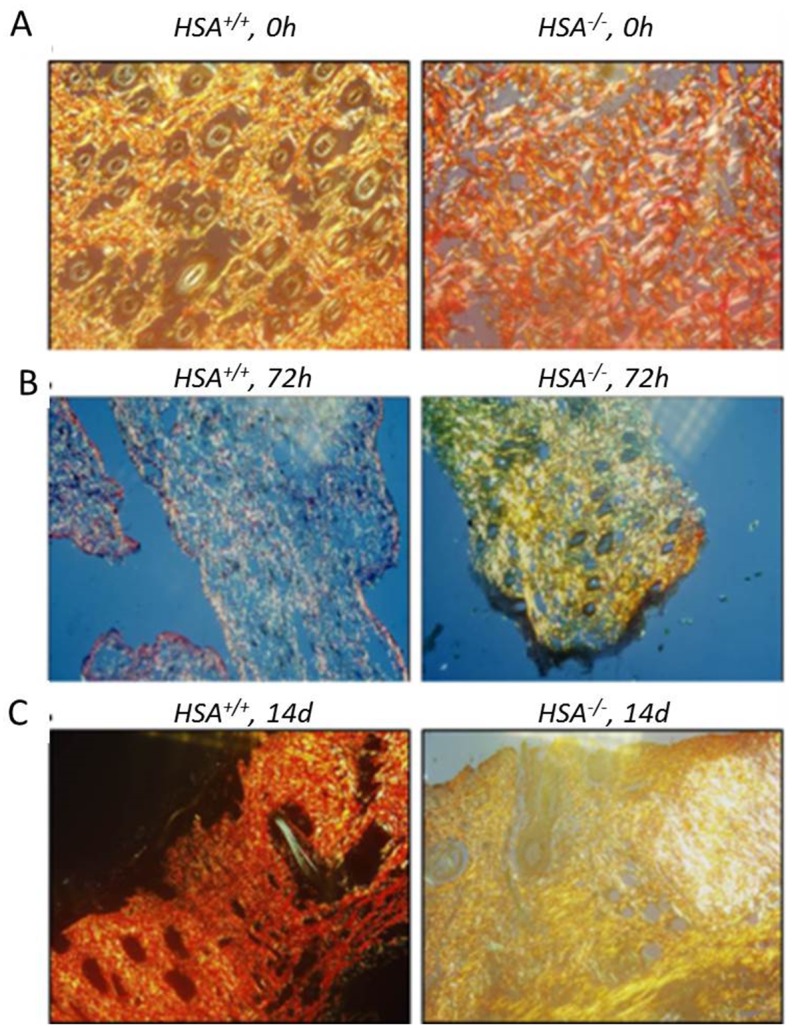
Collagen stains. Tissue sections were stained with NovaUltraTM Picro-Sirius Red stain and visualized on an Olympus AH light microscope at 400× magnification. The left picture in each panel represents the *HSA* ^*+/+*^ mouse while the right is for the *HSA* ^*-/-*^. A. t = 0, time of injury, B. t = 72h, C. t = 14 days. The scoring is detailed in Table 2.

Histologic scoring [19] was based on the degree of cellular infiltration, granulation tissue formation, and re-epithelialization (Table 1). Wounds of WT mice had higher average histologic scores compared to the KO mice. There was a greater degree of cellular infiltration and capillary ingrowth in the *HSA* ^*+/+*^ mice (Fig 3). In addition, the collagen scoring [20] (Table 2) confirmed the IHC staining; the fibers were still separated with complete loss of architecture in the *HSA* ^*-/-*^ mice 72h after the wound was established (Fig 4).

**Table 1:** 

**1–3**	None to minimal cell accumulation. No granulation tissue or epithelial travel				
**4–6**	Thin, immature granulation that is dominated by inflammatory cells but has few fibroblasts, capillaries or collagen deposition. Minimal epithelial migration				
**7–9**	Moderately thick granulation tissue, can range from being dominated by inflammatory cells to more fibroblasts and collagen deposition. Extensive neovascularization. Epithelium can range from minimal to moderate migration				
**10–12**	Thick, vascular granulation tissue dominated by fibroblasts and extensive collagen deposition. Epithelium partially to completely covering the wound				
***wt***			***KO***		
**t = 0**	**t = 72h**	**t = 14 days**	**t = 0**	**t = 72h**	**t = 14 days**
12	4–6	11–12	12	1–3	4–6

**Table 2:** 

	Grade 0	Grade 1	Grade 2	Grade 3
**Collagen**	Collagen arranged in tightly cohesive well demarcated bundles with a smooth dense bright homogeneous polarization pattern with normal crimping	Diminished fiber polarization: separation of individual fibers with maintenance of demarcated bundles	Bundle changes: separation of fibers with loss of demarcation of bundles giving rise to expansion of the tissue overall and clear loss of normal polarization pattern	Marked separation of fibers with complete loss of architecture
	**Wt, KO = t = 0**		**Wt = 72h**	**KO = 72h**

### Production of *HSA*-encoding viruses

**Fig 5:**
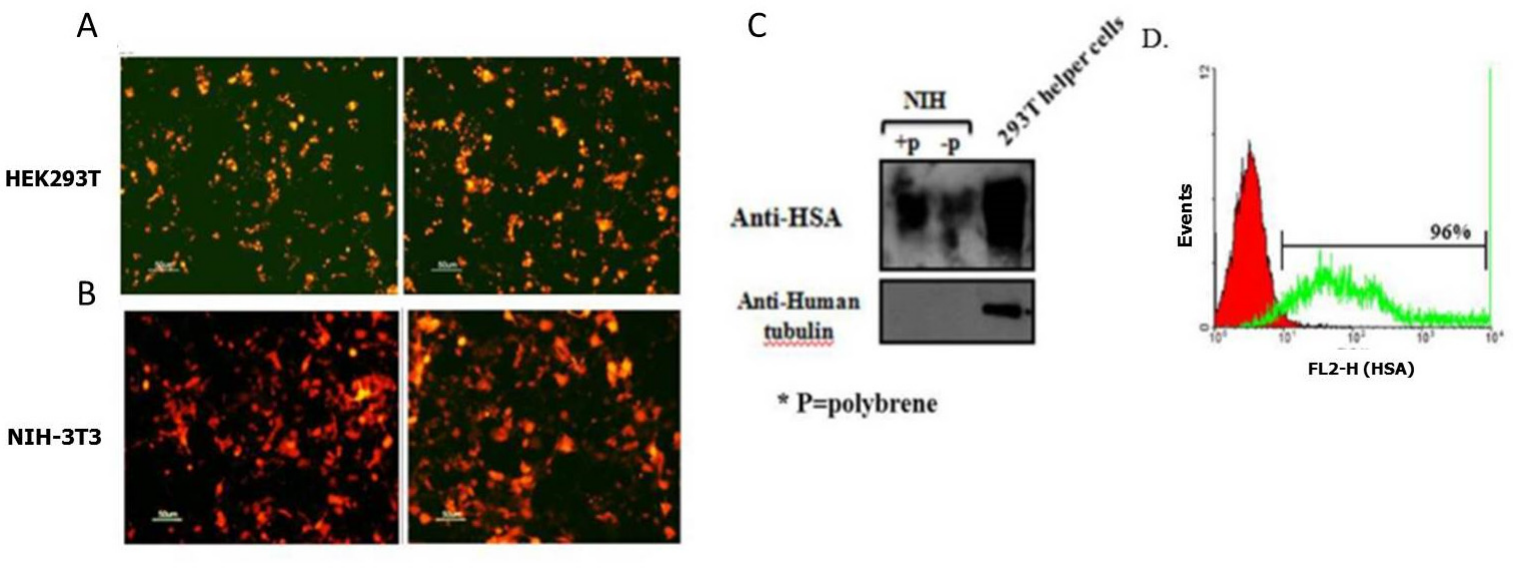
Production of HIV-based viruses for gene delivery. HEK 293T cells were co-transfected with the mentioned plasmids and after 48 h the expression of the transgene was evaluated by the mCherry marker (A). The infectivity of the virions, which was produced by the HEK293T helper cells, was tested on NIH-3T3 *in vitro* by fluorescence microscope (mCherry expression) (B). 72 h after the infection, cell lysates were prepared and 20 μg of total proteins was loaded. HSA was detected with the anti-HSA mAb, M1.69, and then the membrane was reprobed with anti-human tubulin (C). 1x10^6^ infected cells were incubated with 10 μg/ml of M1.69 for 30 minutes at RT. FITC-labeled goat anti-rat antibody was used for the detection of bound antibody. The red curve represents the negative control (secondary antibody alone) and the green curve represents the binding of M1.69 (D).

Three viral-based vectors were used to deliver and express the *HSA* gene in the wounded tissue. First, the expression of the transgene by the helper cells, *in vitro*, was confirmed by monitoring the mCherry fluorescence marker (Fig 5A) and Western blot analysis (Fig 5C). Next, the infectivity of the produced virions was tested *in vitro* on NIH-3T3 cells. Seventy two hours post-infection of NIH-3T3 cells, the expression of the HSA/mCherry proteins was examined and confirmed by fluorescence microscopy (Fig 5B), Western blot (Fig 5C) and FACS analysis (Fig 5D).

### Re-expression of *HSA* restored the healing phenotype

**Fig 6:**
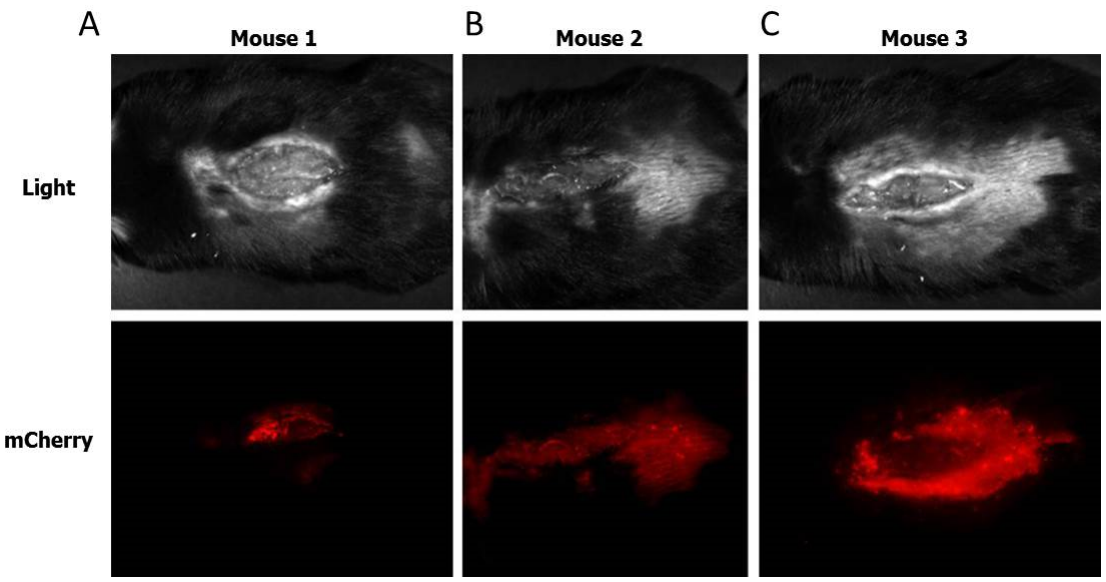
Imaging of the living organism. The viruses were injected or dropped intothe wounded cells immediately after the injury, on t = 0.96 h after injection into the cells on the wound border (A) or dripping(B-C) of the mCherry-encoded viruses (n = 3) onto the wound area, mice were taken to *in vivo* imaging to detect the mCherry expression in the wounded cells using the Cri Maestro device. This imaging tool enables multiplexed *in vivo* fluorescence imaging of small animals with high sensitivity.

Next, we examined whether the expression of HSA in *HSA* ^*-/-*^ mice restores the WT healing capabilities. Firstly, we verified that the viruses infected the cells *in vivo*. To that end, *in vivo* imaging of the wounded tissue in the WT mice 96hoursafter virus injection/dropping confirmed the expression of *mCherry* and HSA in the wounded tissue (Fig 6).

**Fig 7:**
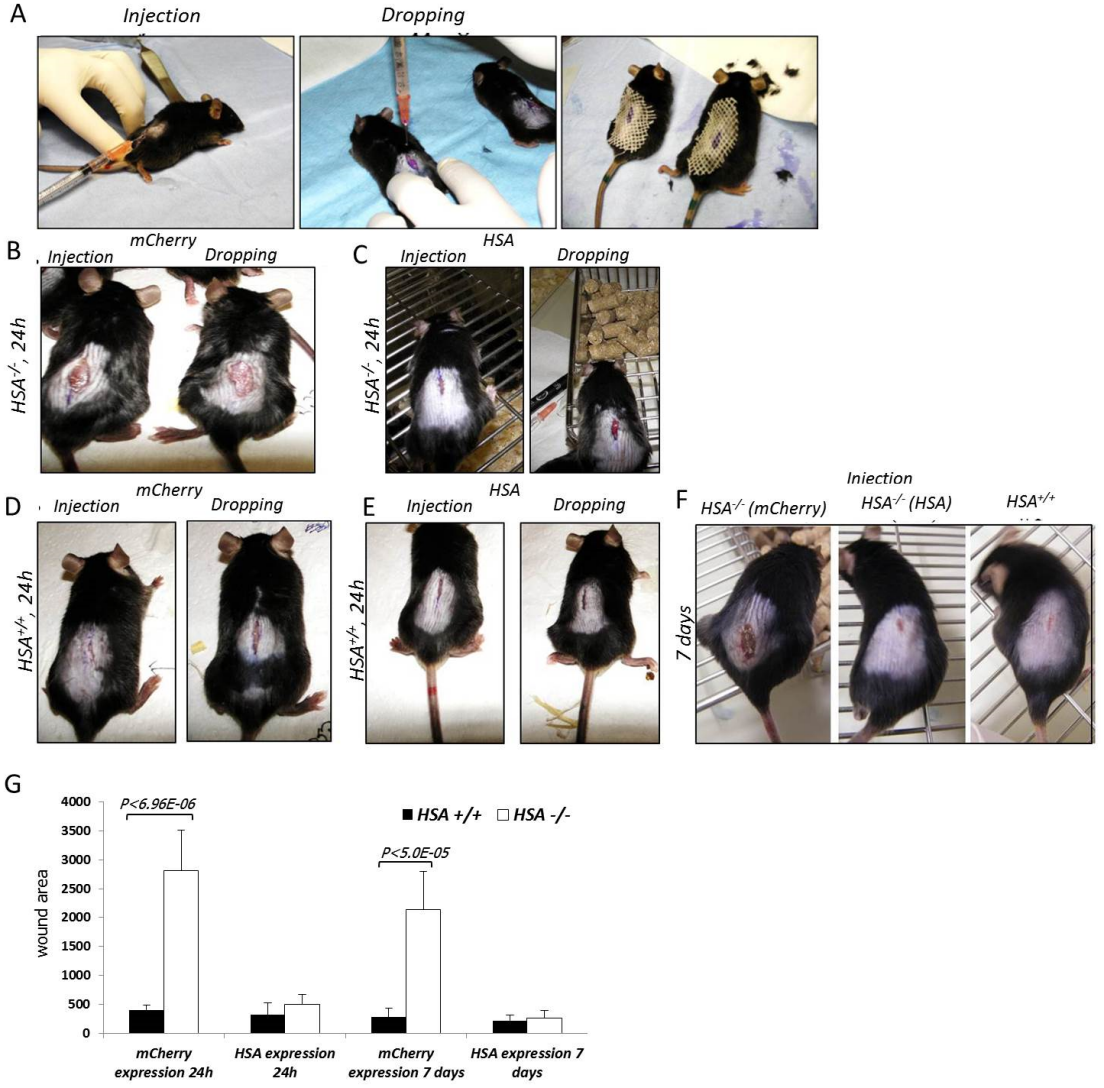
Re-expression of *HSA*. A-The viruses were injected or dropped into the wounded cells on T = 0; B-*HSA* ^*-/-*^ mice 24 h after mCherry-encoded viruses injection (left panel) or dropping (right panel); C-*HSA* ^*-/-*^ mice 24 h after *HSA* encoded viruses injection (left panel) or dropping (right panel); D-*HSA* ^*+/+*^ mice 24 h after *mCherry*-encoded viruses injection (left panel) or dropping (right panel); E-*HSA* ^*+/+*^ mice 24 h after *mCherry*-encoded viruses injection (left panel) or dropping (right panel); F- 7 days after viruses injection; G- A representative plot of the statistical differences, in size of wound area, between the groups.

Then the importance of HSA in the healing process was evaluated by an additional experiment that was performed as described in Table 3. Briefly, mice from each genotype were randomly divided into groups of three mice. Longitudinal incisions of 1.5 cm were made on their back and the HIV-encoding viruses were administrated by injection into the cells on the wound border or by dropping of the viruses into the wounded area. The healing was monitored every day for one week. Re-expression of HSA restores the healing phenotype in the *HSA* ^*-/-*^ mice while the expression of the control mCherry vector had no effect (Fig 7).

**Table 3:** 

	*Number of mice*	*Mice genotype*	*Length of cut*	
**Group 1**	3	*HSA* ^*+/+*^	1.5 cm	Only wound
**Group 2**	3	*HSA* ^*+/+*^	1.5 cm	HIV-mCherry injection
**Group 3**	3	*HSA* ^*+/+*^	1.5 cm	HIV-mCherry dropping
**Group 4**	3	*HSA* ^*+/+*^	1.5 cm	HIV-HSA injection
**Group 5**	3	*HSA* ^*+/+*^	1.5 cm	HIV-HSA dropping
**Group 6**	3	*HSA* ^*-/-*^	1.5 cm	Only wound
**Group 7**	3	*HSA* ^*-/-*^	1.5 cm	HIV-mCherry injection
**Group 8**	3	*HSA* ^*-/-*^	1.5 cm	HIV-mCherry dropping
**Group 9**	3	*HSA* ^*-/-*^	1.5 cm	HIV-HSA injection
**Group 10**	3	*HSA* ^*-/-*^	1.5 cm	HIV-HSA dropping

### Overexpression of *HSA* in WT mice improves wound healing

**Fig 8:**
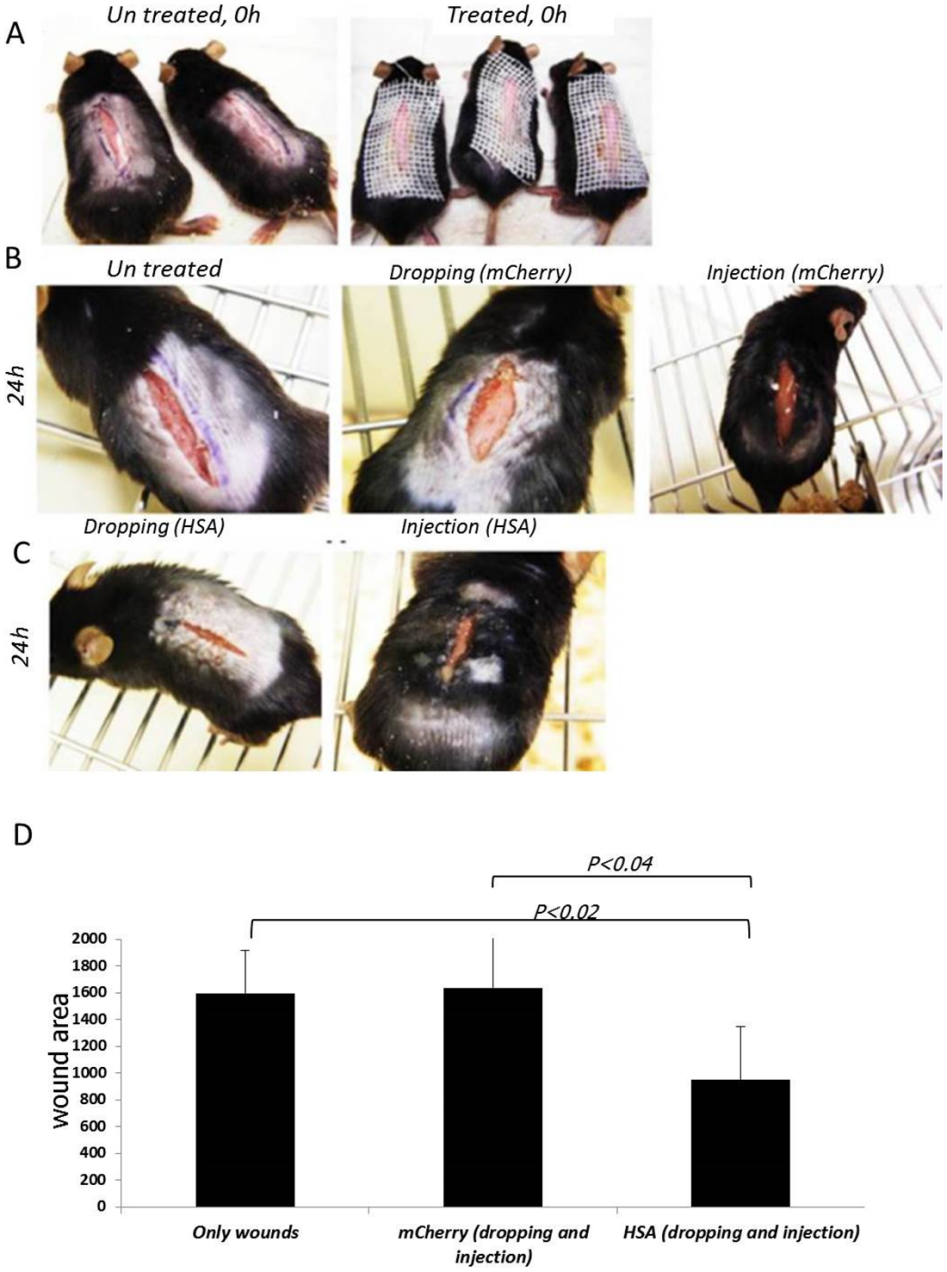
Acceleration of wound healing upon over expression of *HSA*. A-The wounds were infected with the viruses (right panel) or left un-treated (left panel) on T = 0; B- *HSA* ^*+/+*^ mice 24 h after the injury (left panel), 24 h after infection, by dropping, with the mCherry-encoded viruses (middle panel), 24 h after mCherry-encoded viruses injection (right panel); C- *HSA* ^*+/+*^ mice 24 h after infection, by dropping (left panel) or injection (right panel), with the HSA-encoded viruses; D- A representative plot of the statistical differences, in size of wound area, between the groups.

We further evaluated the ability of HSA to improve the wound healing process in normal *HSA* ^*+/+*^WT mice. For this purpose, we enlarged the wound size and the experiment was performed as described in Table 4. Briefly, *HSA* ^*+/+*^ mice were randomly divided into groups of four mice. Longitudinal incisions of4-cm were made on their back and the mCherry or HSA-encoding viruses were administrated twice, 0 and 24 hours post-injury, by injection into the cells in the wound border or by dropping of the viruses into the wounded area and the healing rate was monitored. The results (Fig 8) suggest that overexpression of HSA resulted in faster and improved healing even in *HSA*-expressing mice. Already after 24 hours it was shown that there were differences in the healing rate between mice that were administrated with mCherry and HSA.

**Table 4:** 

	*Wound size*	*Treatment*	*Administration*
**Group 1**	4 cm	NO	
**Group 2**	4 cm	mCherry (T = 0, 24h)	dropping
**Group 3**	4 cm	mCherry (T = 0, 24h)	Injection
**Group 4**	4 cm	HSA (T = 0, 24h)	dropping
**Group 5**	4 cm	HSA (T = 0, 24h)	Injection

**Fig 9:**
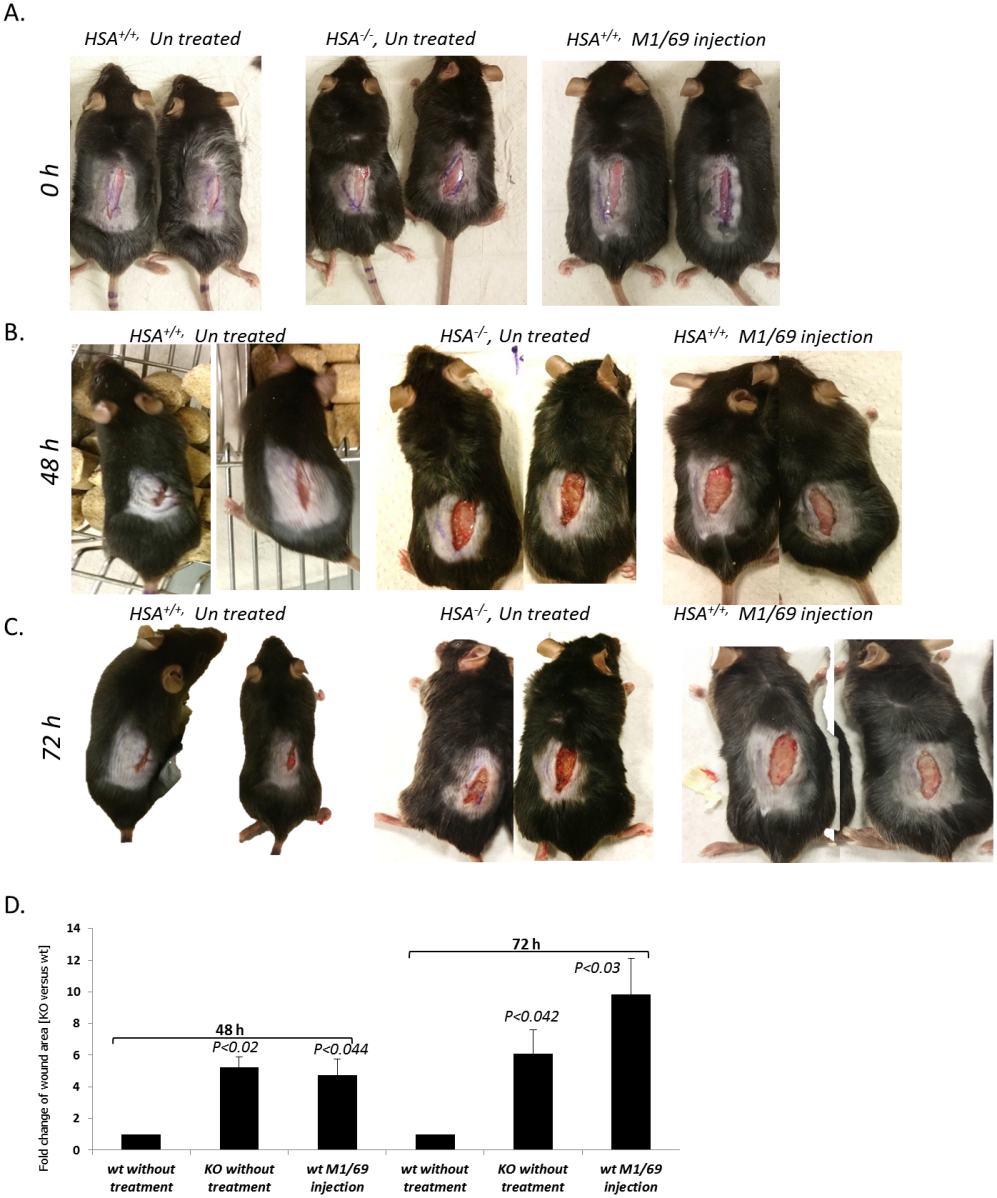
Delayed wound healing upon down regulation of *HSA* expression. A-The wounds were treated with anti-HSA antibody (right panel) or left un-treated (left and middle panel) on T = 0; B- *HSA* ^*+/+*^ (left panel) and *HSA* ^*-/-*^ (middle panel) mice 48 h after the injury and *HSA* ^*+/+*^ mice 48 h after antibody injection (right panel); C- B- *HSA* ^*+/+*^ (left panel) and *HSA* ^*-/-*^ (middle panel) mice 72 h after the injury and *HSA* ^*+/+*^ mice 72 h after antibody injection (right panel); D- A representative plot of the statistical differences, in size of wound area, between the groups. Student t-test was calculated in each time point between the tested group and the wt without treatment group.

In order to confirm the importance of CD24/HSA in the healing process, a complementary experiment was performed. *HSA* ^*+/+*^ mice were randomly divided into groups of three mice. Longitudinal incisions of 2 cm were made on their back and the anti-HSA M1/69 mAb (10 mg/kg) was administrated immediately post-injury by injection into the cells in the wound border. The results suggest that antibody-mediated reduction of HSA expression level caused to slower and non-homogenous healing in HSA-expressing mice (Fig 9).

### The improvement of healing by *HSA* expression does not depend on the mice strain

**Fig 10:**
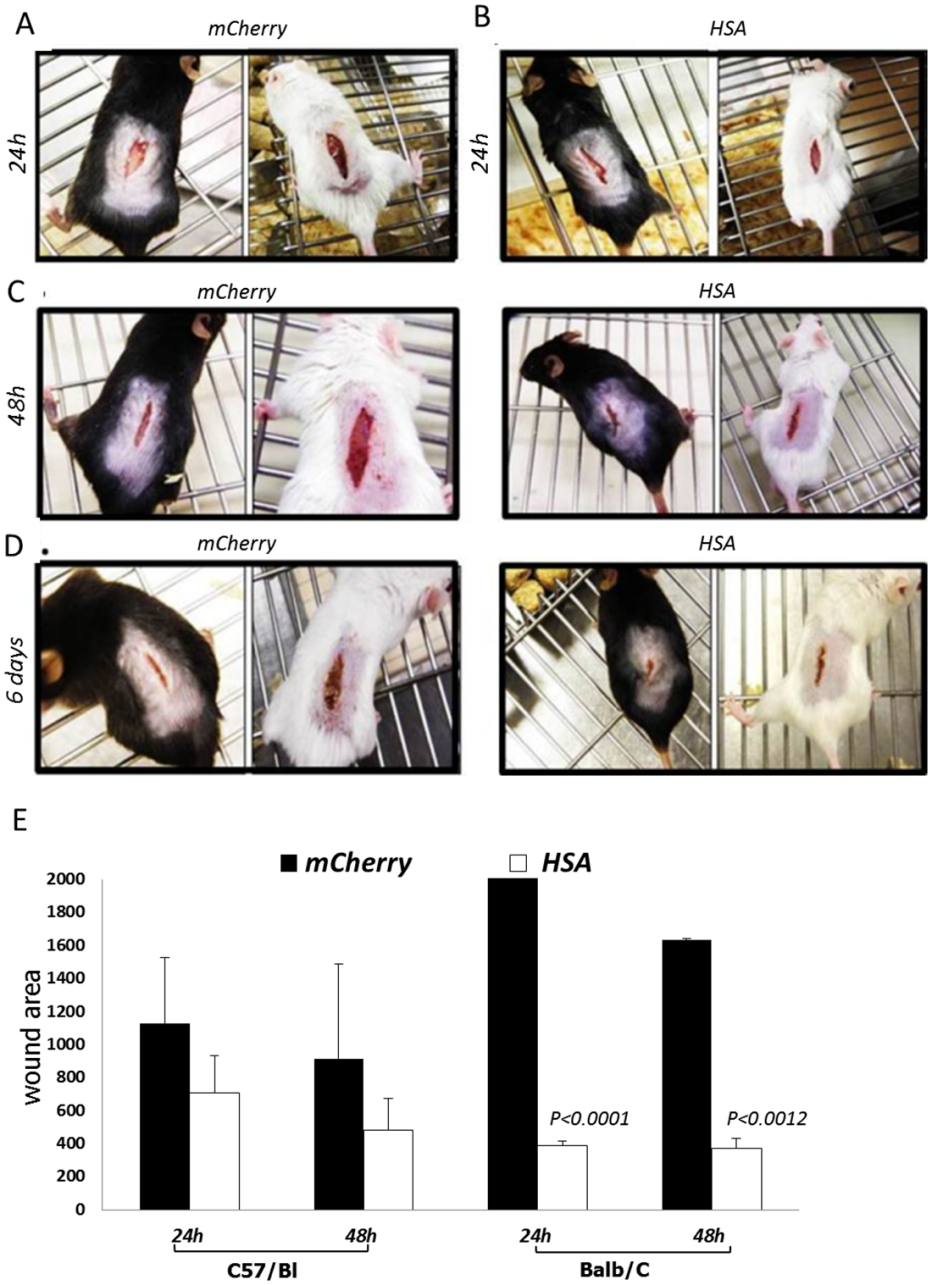
The rapid healing does not depend on mice strain. Four groups of four mice were used in this study. Two of them were of C57/Bl while the other two were of Balb/c. The mice in one group from each strain were injected with the HSA-encoded viruses while the other group with the mCherry-encoded viruses. A- *HSA* ^*+/+*^ mice 24 h after *mCherry*-encoded viruses injection; B- *HSA* ^*+/+*^ mice 24 h after *HSA*-encoded viruses injection; C- *HSA* ^*+/+*^ mice 48 h after *mCherry*-encoded viruses (the two pictures on the left) and *HSA*-encoded viruses injection (the two pictures on the right); D- *HSA* ^*+/+*^ mice 6 days after mCherry-encoded viruses (the two pictures on the left) and *HSA*-encoded viruses injection (the two pictures on the right); E-A representative plot of the statistical differences, in size of wound area, between the groups. Student t-test was calculated in each time point between the HSA treated group and the mCherry treated group.

In order to examine if the above phenotype depends on the mice strain, we compared between Balb/C and C57Bl/6JWTmice. Eight mice from each strain were randomly divided to two groups of four mice. One group of each strain was injected with *HSA*-encoding viruses, immediately after the injury, while the second group with the control vector. According to our observations, the wound healing was improved regardless of the mice strain as can be demonstrated in Fig 10.

## Discussion

Our study shows that HSA in mice plays an important role in wound healing. Restoration of the protein restores impaired wound healing back to normal. Moreover, forced expression of HSA enhances the healing process.

CD24 expression has long been correlated with pathologies associated with tumor cell migration and invasion [21–23]. The exact function and mechanism of action of CD24 is mostly unknown; however, it is thought to be involved in a variety of processes such as cell proliferation and it also changes the adhesive properties of tumor cells by promoting their adhesion to P-selectin, fibronectin, collagens I and IV, and laminin [22]. Tumors have been previously described as wounds that do not heal. Wound healing and cancer have similar properties of cellular behavior. They are both characterized by increased cell proliferation, survival, invasion and migration (e.g. angiogenesis and metastasis), remodeling of extracellular matrix, new blood vessel formation, and modulation of blood coagulation. The molecular programs in normal wound healing and those in tumor progression and metastasis were found to be similar [24,25], [26]. Data from our laboratory suggest that overexpression of CD24 is associated with increased cell migration in wound healing assays *in vitro* (Naumov et al., 2014)[27] showed that cells that overexpress CD24 proliferate faster, and increase and cell motility, saturation density, plating efficiency, and growth in soft agar. They also produce larger tumors in nude mice as compared to the parental cells. In addition, We have shown, Shapira et al., JBC 2011, [28]that induced expression of CD24 in a dose- and time-dependent fashion (using the Tet-On system), led to an increased proliferation which was inhibited by mAb to CD24. *In vivo*, significantly larger tumors were developed in tetracycline-fed mice compared to the control group. Taking all of this together with the data presented here, we suggest that increased expression of CD24 promotes growth pathways and thus accelerates and improves the healing of wounded tissue. *HSA*-deficient mice have delayed healing. When the wounds were stitched, the stitches did not hold, suggesting that CD24 is required. In most cases, the healing process was not only delayed but it failed to progress through the normal stages of healing, and resulted in an inflamed scar. Most importantly, re-expression of HSA in *HSA*-deficient mice, by lentiviruses, restores the wound closure rate in WT mice.

Topical administration of anti-HSA antibodies confirms the genotypic observations of the importance of HSA in normal wound healing.

Interestingly, preliminary data emerging from mouse models of bone (tibia fracture model) fracture and tooth extraction strengthen the importance of CD24 in fracture and tooth extraction healing (Arber *et al*., unpublished data).

Our hypothesis is that CD24 plays a key role in cell proliferation, migration and adhesion of healthy cells to the damaged area to restore normal tissue. Increased expression of the CD24 protein may represent a novel clinical intervention strategy to accelerate the healing of debilitating acute or chronic wounds in patients.
